# Cost-utility analysis of an implantable cardioverterdefibrillator for the treatment of patients with ischemic or non-ischemic New York Heart Association class II or III heart failure in Colombia

**DOI:** 10.7705/biomedica.4235

**Published:** 2019-09-01

**Authors:** Sara Atehortúa, Juan Manuel Senior, Paula Castro, Mateo Ceballos, Clara Saldarriaga, Nelson Giraldo, Guillermo Mora

**Affiliations:** 1 Departamento de Economía, Facultad de Ciencias Económicas, Universidad de Antioquia, Medellín, Colombia Universidad de Antioquia Departamento de Economía Facultad de Ciencias Económicas Universidad de Antioquia Medellín Colombia; 2 Departamento de Cardiología Clínica y Cardiología Intervencionista, Facultad de Medicina, Universidad de Antioquia, Medellín, Colombia Universidad de Antioquia Departamento de Cardiología Clínica y Cardiología Intervencionista Facultad de Medicina Universidad de Antioquia Medellín Colombia; 3 Grupo de Rehabilitación en Salud, Facultad de Medicina, Universidad de Antioquia, Medellín, Colombia Universidad de Antioquia Grupo de Rehabilitación en Salud Facultad de Medicina Universidad de Antioquia Medellín Colombia; 4 Grupo de Economía de la Salud, Facultad de Ciencias Económicas, Universidad de Antioquia, Medellín, Colombia Universidad de Antioquia Grupo de Economía de la Salud Facultad de Ciencias Económicas Universidad de Antioquia Medellín Colombia; 5 Instituto de Evaluación Tecnológica en Salud, Bogotá, D.C., Colombia Instituto de Evaluación Tecnológica en Salud BogotáD.C Colombia; 6 Grupo de Epidemiología Clínica, Facultad de Medicina, Universidad de Antioquia, Medellín, Colombia Universidad de Antioquia Grupo de Epidemiología Clínica Facultad de Medicina Universidad de Antioquia Medellín Colombia; 7 Departamento de Medicina Interna, Facultad de Medicina, Universidad Nacional de Colombia, Bogotá, D.C., Colombia Universidad Nacional de Colombia Departamento de Medicina Interna Facultad de Medicina Universidad Nacional de Colombia BogotáD.C Colombia; 8 Servicio de Electrofisiología, Fundación Santa Fe de Bogotá, Bogotá, D.C., Colombia Servicio de Electrofisiología Fundación Santa Fe de Bogotá BogotáD.C Colombia

**Keywords:** Heart failure, defibrillators, implantable, death, sudden, cardiac, cost-benefit analysis, Colombia, insuficiencia cardíaca, desfibriladores, muerte súbita cardíaca, análisis costo-beneficio, Colombia

## Abstract

**Introduction:**

The use of an implantable cardioverter-defibrillator reduces the probability of sudden cardiac death in patients with heart failure.

**Objective:**

To determine the cost-utility relationship of an implantable cardioverterdefibrillator compared to optimal pharmacological therapy for patients with ischemic or nonischemic New York Heart Association class II or III (NYHA II-III) heart failure in Colombia.

**Materials and methods:**

We developed a Markov model including costs, effectiveness, and quality of life from the perspective of the Colombian health system. For the baseline case, we adopted a time horizon of 10 years and discount rates of 3% for costs and 3.5% for benefits. The transition probabilities were obtained from a systematic review of the literature. The outcome used was the quality-adjusted life years. We calculated the costs by consulting with the manufacturers of the device offered in the Colombian market and using national-level pricing manuals. We conducted probabilistic and deterministic sensitivity analyses.

**Results:**

In the base case, the incremental cost-effectiveness ratio for the implantable cardioverter-defibrillator was USD$ 13,187 per quality-adjusted life year gained. For a willingness-to-pay equivalent to three times the gross domestic product per capita as a reference (USD$ 19,139 in 2017), the device would be a cost-effective strategy for the Colombian health system. However, the result may change according to the time horizon, the probability of death, and the price of the device.

**Conclusions:**

The use of an implantable cardioverter-defibrillator for preventing sudden cardiac death in patients with heart failure would be a cost-effective strategy for Colombia. The results should be examined considering the uncertainty

## Resumen

**Introducción.:**

El uso del desfibrilador cardioversor implantable reduce la probabilidad de muerte súbita en pacientes con insuficiencia cardíaca.

**Objetivo.:**

Determinar la relación de costo-utilidad de un desfibrilador cardioversor implantable comparado con la terapia farmacológica óptima para pacientes con insuficiencia cardíaca isquémica o no isquémica de clase II o III según la *New York Heart Association* (NYHA II-III) en Colombia.

**Materiales y métodos.:**

Se desarrolló un modelo de Markov que incluyó los costos, la efectividad y la calidad de vida desde la perspectiva del sistema de salud colombiano. Para el caso de base, se adoptó un horizonte temporal de 10 años y una tasa de descuento de 3 % para los costos y de una 3,5 % para los beneficios. Las probabilidades de transición se obtuvieron de una revisión sistemática de la literatura. Los años de vida ajustados por calidad se usaron como resultado de salud. Para estimar los costos unitarios se usaron manuales tarifarios nacionales y, para los costos del dispositivo, se consultó a los fabricantes que lo comercializan en el mercado colombiano. Se hicieron análisis de sensibilidad probabilísticos y determinísticos.

**Resultados.:**

En el caso base el costo adicional por año de vida ajustado por calidad ganado con el desfibrilador cardioversor implantable fue de USD$ 13,187. Usando un umbral de tres veces el producto interno bruto per cápita como referencia (USD$ 19.139 en 2017), el dispositivo sería costo-efectivo para el sistema de salud colombiano. Sin embargo, este resultado depende del horizonte temporal, de la probabilidad de muerte y del precio del dispositivo.

**Conclusiones.:**

El uso de un desfibrilador cardioversor implantable sería una estrategia costo-efectiva para Colombia, aunque los resultados deben examinarse teniendo en cuenta la incertidumbre.

Heart failure is an important medical, social, and economic problem. More than 37.7 million people worldwide suffer from this condition and in 2009 alone, 870,000 new cases per year occurred from 2005 to 2011 were registered in the United States ^1^^,^^2^. Heart failure is associated with a high financial burden, consuming approximately 2% of the total health costs in developed countries ^3^. The costs of care for this condition totaled 30.7 billion dollars for the United States health system in 2012 ^4^.

Despite the scarce epidemiological data in the majority of developing countries, it is estimated that the prevalence of heart failure among the adult population ranges from 2 to 3% and tends to increase with age ^5^. In Colombia, there are no accurate disease prevalence registries, but it is known that 40 % of hospitalizations due to cardiovascular diseases are associated with heart failure ^6^.

Although optimal pharmacological therapy (OPT) is highly recommended for the management of patients with heart failure, it is not always an effective alternative for the prevention of sudden cardiac death ^7^, one of the principal causes of death in patients with this condition. For this reason, the implantable cardioverter-defibrillator (ICD) was developed; it is a device that has been shown to reduce the likelihoods of death from all causes and of sudden cardiac death in patients with ischemic and non-ischemic heart failure and left ventricular systolic dysfunction (LVSD) ^8^^,^^9^.

The implantation of an ICD is increasingly common in Colombia, and its high cost could imply an important increase in the financial burden for the health system. The objective of this study was to determine the cost-effectiveness relationship between the use of an ICD and OPT compared to the OPT alone to avoid sudden cardiac death in patients with heart failure from the perspective of the Colombian health system.

## Materials and methods

This cost-utility analysis was conducted from the perspective of the Colombian health system including the direct care costs financed by the health system. The study population was composed by persons with NYHA II-III functional heart failure, an ejection fraction < 35%, LVSD, without a previous history of sudden cardiac death, and with ischemic or non-ischemic cardiomyopathy. The analysis was conducted in this group of people because they are at greater risk of sudden cardiac death and would, therefore, benefit most from an ICD implantation ^8^^,^^9^. The initial age of our hypothetical cohort was 60 years. For the base case, a time horizon of 10 years and discount rates of 3% for costs and 3.5% for benefits were assumed according to the recommendations of the methodological guide for developing economic evaluations within Colombia’s clinical practice guidelines ^10^.

**Figure 1:**
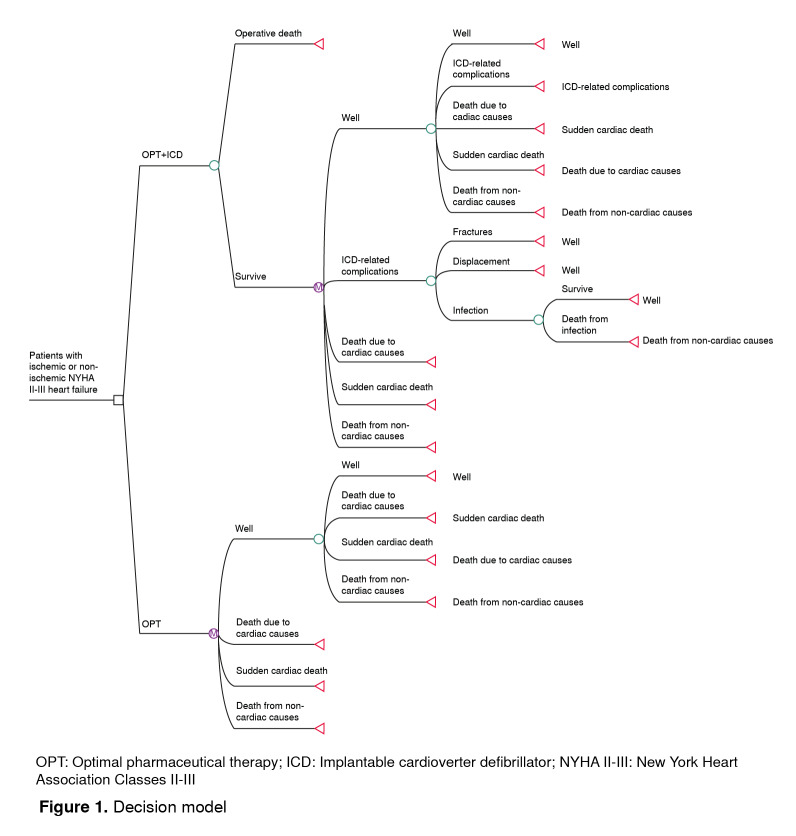
Decision model

To estimate the expected costs and benefits from each alternative, a decision tree that simulated outcomes associated with the ICD surgical implantation phase and a Markov model with annual cycles were employed to simulate short and long-term effects (figure 1). Patients who underwent an ICD implantation could either experience post-operatory death (death occurring during the first 30 days after the surgical procedure) or they could survive. They could also enter into one of the stages that make up the Markov model: Suffering sudden cardiac death, non-sudden cardiac death, or death due to non-cardiac causes; they could experience no event, or they could experience some ICD-related complication. Fractures, displacement, and severe infection were the ICD related complications considered because they usually are more frequent and imply significant cost increases. For severe infections, the possibility of the patient dying from such cause was considered. For the OPT alternative, the same outcomes were considered, but post-operatory ICD-related death and complications were excluded.

The effectiveness outcome employed was the quality-adjusted life years (QALY). This outcome focuses on patients and combines the years of life gained and the health-related quality of life score for patients with heart failure.

Transition probabilities were extracted from clinical studies found in a systematic review of the literature in Medline, EMBASE, and Cochrane Library databases included in a Colombian clinical practice guideline for heart failure (CPG-HF) ^11^. The search terms used were heart failure, cardiac failure, myocardial failure, heart decompensation, NYHA, defibrillators, implantable, and cardioverter-defibrillator.

From the available evidence, we used a meta-analysis aggregating evidence from various clinical trials ^8^ conducted in ischemic and non- ischemic patients, which focused only on ICD (without including the effects of cardiac resynchronization therapy) and excluded heterogeneous trial results. To calculate the probability of all causes of death and sudden cardiac death in the ICD branch of the tree, we multiplied the probability of death in the OPT branch (baseline) by the relative risk (RR) reported in the meta-analysis (0.73 and 0.4, respectively). Using this procedure, we obtained the probabilities reported in table 1. The probability of operative death used in the base case was also obtained from this meta-analysis ^8^.

**Table 1:** Model parameters, base case estimates, and data sourcesOPT: Optimal pharmaceutical therapy; ICD: Implantable cardioverter defibrillator; N/A: Not applicable

Alternative	Variable	Base case estimates	Distribution parameters (alpha, beta, min-max)	Data source
Probabilities				
ICD	Operative death	0	α=0; β=2,774	(8)
	All-cause mortality	0.178	α=385; β=1,790	(8)
	Sudden cardiac death	0.037	α=110; β=2,825	(8)
	ICD-related complications	0.062	α=169; β=2,554	(8)
	Devise movement	0.5	α=16; β=16	(8)
	Infection	0.2	α=10; β=40	(8)
	Death from infection	0.21	α=1.93; β=7.27	(12, 13)
OPT	All-cause mortality	0.284	α=604; β=1,522	(8)
	Sudden cardiac death	0.108	α=317; β=2,605	(8)
Utility weight				
	Well (equal to both alternatives)	0.845	α=6.164; β=1.131	(14)
	ICD-related complications	0.75	N/A	(15, 16)
Medical and procedure related costs				
OPT	Medications, consultations, and laboratory tests	313.04	282.08 - 343.95	
ICD	Device price	7,259.46	6,539.44 - 7,793.12	
	Implant procedure	300.28	289.80 - 337.59	
	Post-implant hospitalization	73.39	70.11 - 84.01	
	Major infection	194.17	184.57 - 223.73	
	Fracture/Displacement	187.83	180.60 - 213.83

OPT: Optimal pharmaceutical therapy; ICD: Implantable cardioverter defibrillator; N/A: Not applicable

The probability of non-sudden cardiac death was obtained by subtracting all-cause mortality from the sudden cardiac death and death from other non- cardiac causes. Age-adjusted probability of cardiac death due to all causes was taken from the statistics of the Colombian *Departamento Administrativo Nacional de Estadísticas* (DANE). For the life tables by age groups, we considered mortality due to heart failure (CIE-10 303 and 306 codes) and mortality from other causes (those reported).

Given that the probability of death due to infection was not reported in any of the clinical trials available, we used the information reported in economic evaluations of ICD use in patients with heart failure ^12^. The model assumed that the benefits of the ICD found in the follow-up of the clinical trials were constant and could be extrapolated to all time frames proposed. However, considering age-adjusted probabilities, we considered an increase in the mortality rate over time for a more realistic scenario.

For all transition probabilities of the model, beta distributions with α and β parameters were constructed based on population data. For the probability of death by infection, α and β parameters were constructed from the mean and standard deviation of the estimates.

To determine utility weights for the model states, we reviewed the Tufts University’s Cost-Effectiveness Analysis (CEA) Registry to obtain those used in heart failure clinical trials, as well as multiple estimates from cohort studies. For the base case, we chose data calculated in the Multicenter Automatic Defibrillator Implantation Trial-Cardiac Resynchronization Therapy (MADIT- CRT) clinical trial for the “well” state ^13^ due to its proximity to the population, the alternatives analyzed, and the detail of the data presented while for the ICD “complications” state, the calculation was taken from the literature ^14^^,^^15^. It was assumed that ICD implantation did not change the quality of life of the patients who remained in the well state of the Markov model. The weights used in the base case are shown in table 1.

The direct medical costs associated with each alternative and branch of the model were calculated based on the identification and measurement of the resources consumed. This calculation was performed through the construction of a type-case according to the review of the CPG-HF clinical recommendations and from health care protocols of a Colombian hospital. This type-case was validated and modified based on an informal consensus of general internists and cardiologists who are experts in the field ^11^. Details regarding the units of measurement and the frequency of resources included can be found in the CPG-HF ^11^.

To calculate the cost of the procedures, we used the Colombian *Instituto de Seguros Sociales* tariffs from 2001 with a 30% adjustment for the base case and 25% and 48% adjustments for the minimum and maximum values, respectively. According to the methodological guide for developing economic evaluations in Colombia, these adjustments update the costs of the procedures to the current conditions of the Colombian market ^10^.

The unit prices of medications were calculated with the information reported in the institution-laboratory channel of the Colombian pricing and medication information system for 2017. The minimum, mean, and maximum prices of each presentation correspond to the weighted estimates of the different medication presentations, which include both generic and brand name drugs.

The price of the ICD was obtained from the quotations of two companies that manufacture and market this type of device in the country. In the base case, we considered the average prices of the two types of devices (single- and dual-chamber). Supposedly, the device needs a replacement every 5 years and the costs associated with this procedure correspond exclusively to the implant of the device battery and does not include the cost of the electrodes and wires. The average ICD price was calculated in USD$ 7,259.46 with a minimum price of USD$ 6,539.44 and a maximum of USD$ 7,793.12.

To determine the costs associated with the OPT, we included the standard medical management with three types of basic medications: Angiotensin- converting enzyme inhibitors (ACEI) or angiotensin II receptor antagonists (ARA II), beta-receptor blockers, and diuretics as reviewed by specialists, as well as some laboratory exams and diagnostic aids. The cost of the OPT used in the base case is shown in table 1.

For the ICD-associated costs, we also considered OPT management, the price of the device (including the cardioverter-defibrillator, the electrodes, and the wires), and the costs of the implantation surgery and the subsequent recovery hospitalization. Details regarding the specific resources included in each component can be found in the CPG-HF ^11^. The prices of all of the elements making up the total cost of the ICD considered in the base case are shown in table 1.

In addition to the costs of the comparison alternatives, we considered the resources consumed in the treatment of ICD complications. In the initial emergency procedures, we included care for severe infection, antibiotic treatment, consultation with specialists, and the price of the electrode removal surgery price and replacement with a new device (which included the implantation procedure and subsequent hospitalization). Electrode displacement or fracture included emergency care, consultations, and repositioning procedures. The additional cost associated with each complication for the base case is shown in table 1 considering that in the case of severe infection, the cost of a new ICD device should be added.

To address uncertainty, we conducted deterministic sensitivity analyses of the cost of the device and the ICD replacement time, as well as of the probability of death due to all causes over time. Additionally, we did a tornado analysis to assess the impact of all the variables in the results. Finally, we performed a subgroup analysis for ischemic and non-ischemic patients using the different data provided by Theuns, *et al.*^8^^).^

We also performed a probabilistic sensitivity analysis with 10,000 Monte Carlo simulations using beta distributions for transition probabilities and utility weights and uniform distributions for costs. The distribution parameters for the inputs of the model are presented in table 1.

To determine the relationship between costs and QALY, we used the incremental cost-effectiveness ratio (ICER), which was compared with a cost-effectiveness threshold. Although the explicit definition of the threshold is a controversial topic, this study followed the recommendation of the World Health Organization of a threshold between 1 and 3 times the country’s per capita gross domestic product (GDP) ^16^. According to the official data from Colombia’s central bank, the threshold for 2017 ranged between USD$ 6,308 and USD$ 19,139. The model and the statistical analyses were done using TreeAge Pro 2013 (TreeAge Software Inc., Williamstown, MA). Costs in US dollars (USD) were calculated using the representative exchange rate for the Colombian market in 2017 reported by the country’s central bank in COP$ 2,951.32 per dollar.

## Results

In the base case and over a time horizon of 10 years, the cost for the ICD per QALY gained was USD$ 13,187. This result led to the conclusion that the device would be a cost-effective alternative for the Colombian health system, as it did not exceed USD$ 19,139, i.e., the equivalent of three times the Colombian per capita GDP for 2017. However, when we considered a time horizon of 5 years, the ICER reached USD$ 20,569, which is higher than the threshold used (table 2).

**Table 2:** Cost-effectiveness of OPT compared to OPT plus ICD

Strategy	Costs (USD)	Incremental cost	QALY	Incremental QALY	ICER
Base-case					
OPT	974		2.6056		
OPT + ICD	10,992	10,018	3.3653	0.7596	13,187
Sensitivity analysis - time horizon (5 and 20 years)					
OPT	890		2.3859		
OPT + ICD	10,322	9,432	2.8445	0.4585	20,569
OPT	987		2.6385		
OPT + ICD	11,194	10,207	3.5225	0.8840	11,546
Analysis by subgroups (ischemic and non-ischemic)					
OPT	973		2.6029		
OPT + ICD	11,314	10,341	3.5449	0.9420	10,977
OPT	1,243		3.3146		
OPT + ICD	12,252	11,008	4.0488	0.7342	14,992

OPT: Optimal pharmaceutical therapy; ICD: Implantable cardioverter defibrillator; ICER: Incremental costeffectiveness ratio; QALY: Quality-adjusted life years

When considering a device-replacement time of 7 years, the ICER considerably decreased to USD$ 11,865. Furthermore, assuming that all variables kept constant over time but modifying the ICD price, we still found it to be a cost-effective alternative provided that its cost did not exceed USD$ 10,685.

When the cost-effectiveness relationship of the ICD was analyzed for ischemic patients, we calculated a cost of USD$ 10,977 per QALY gained while for non-ischemic patients this value increased to USD$ 14,992. Considering a threshold of USD$ 19,139, we concluded that the ICD was a cost-effective alternative for both types of patients, although a better relationship between costs and QALY was found for ischemic patients (table 2).

**Figure 2:**
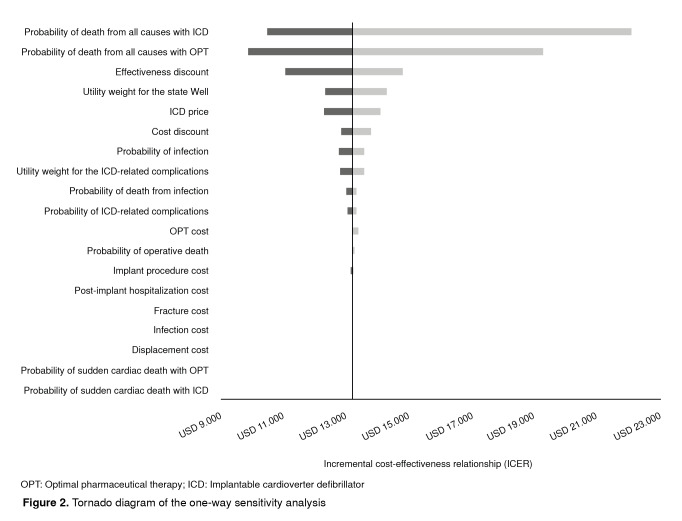
Tornado diagram of the one-way sensitivity analysis

**Figure 3:**
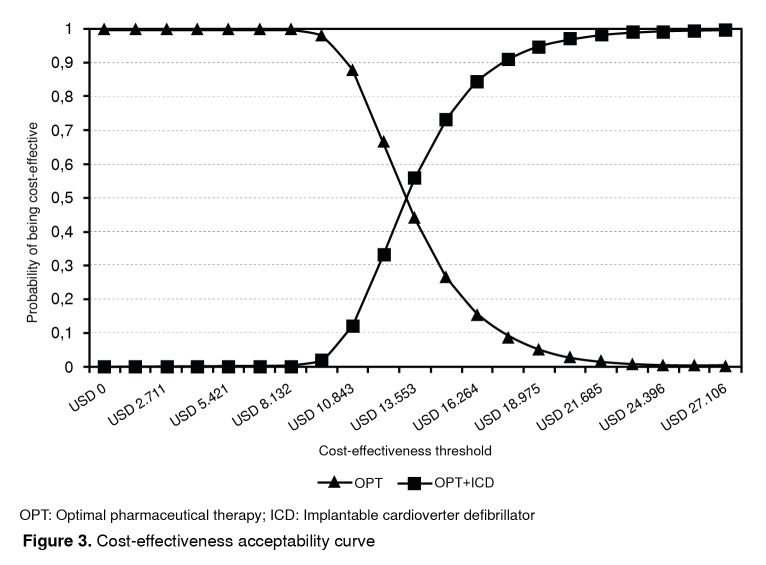
Cost-effectiveness acceptability curve

The tornado analysis allowed us to conclude that the probability of death variables was more relevant than others to change the result obtained in the base case (figure 2). Finally, in the probabilistic sensitivity analysis, if the willingness to pay per QALY equaled USD$ 19,139, the probability that the ICD would be cost-effective was 95.1% (figure 3).

## Discussion

The results of this economic analysis suggest that the ICD would be a cost-effective alternative for the Colombian health system in the treatment of patients with both ischemic and non-ischemic heart failure, an ejection fraction <35%, LVSD, and functional class NYHA II-III over a time horizon of 10 years. This was consistent as long as its price did not exceed USD$ 10,685. The decision also depended largely on the threshold decided upon, as the base case results were sensitive to this choice.

These results are similar to other economic evaluations published in developed countries, which have found that although the ICD implies an increase in cost for the health system, it is cost-effective at a patient- population level as its use significantly reduces mortality ^14^^,^^15^^,^^17^^,^^18^. However, the majority of the economic evaluations published have beendesigned in the context of developed countries and, as such, they respond to different characteristics from those in Colombia, which make them not completely comparable.

To the best of our knowledge, only two evaluations in the Latin American context have been published ^12^^,^^19^. The first found that the ICD was not cost-effective for the Brazilian health system but it had a better cost- effectiveness relationship for patients at greater risk of sudden cardiac death. The second showed heterogeneous results depending on the cohort of patients and the specific Argentinian health sub-system.

The main difference between our results and those from these two papers relies on the effectiveness source used. While the Brazilian study used data from a local cohort of patients and a meta-analysis, our research resorted to data from a recent international aggregate study, which directly impacts the cost-effectiveness estimation. Similarly, the Argentinian study considered three randomized clinical trials independently and not aggregated.

Recently, the Danish Study to Assess the Efficacy of ICD in Patients with Non-Ischemic Systolic Heart Failure on Mortality (DANISH) reported the lack of any survival benefit of ICD in patients with non-ischemic cardiomyopathy, which could alter the results of cost-utility analyses ^20^^,^^21^. However, the study did not address the mortality effects in non-ischemic cardiomyopathy patients who only had ICD. On the other hand, a new meta-analysis including the DANISH trial found a decrease in mortality in this population ^22^^,^^23^.

Some of the limitations of this study are related to the probabilities of death, which are the more sensitive variables for cost-effectiveness conclusions. Estimating probabilities for the Colombian population could be difficult because we would need information from a randomized clinical trial currently not available. This forced us to use probabilities obtained from the best clinical evidence available. A broad methodological discussion about clinical evidence can be found in the CPG-HF recommendations ^11^.

Another limitation is the lack of weighted utility estimates for Colombia. The results could vary if the health estimates to be included in the Markov model were different for the Colombian population. Estimating valid utility values for the local setting is a research effort that could make the cost utility analysis more robust in the present study and in future ones conducted in the country, as the QALY constructed would correspond to the Colombian population.

Concerning costs, one limitation that should be mentioned stems from the fact that the ICD pricing data were obtained via direct quotation from two of the four companies that manufacture and distribute the device in the Colombian market, as there are no institutional registries for determining whether only these companies effectively sell the devices. More importantly, no systematic information on the prices that care providers and insurers pay were available to establish whether differences in contracts or operating margins could modify the prices actually paid compared to those in the market.

Assuming that five-year replacement costs exclude those costs of the electrodes and other accessories could be a limitation, as they could require replacement in some cases either because they no longer work adequately or due to complications in the procedure. Unfortunately, there are no data in Colombia to establish the replacement percentage required for electrodes and other accessories. We hope such a percentage would be minimum, given that the elective change rate varies between 1% and 5% depending on the type of electrode used ^24^.

Another possible limitation lies on the OPT costs, as we used the weighted average per molecule for an important group of medications in the base case. For renin-angiotensin-aldosterone system-blocking medications, for example, we used the weighted average, which includes both ARA IIs and ACE inhibitors, although these are indicated only for patients who do not tolerate the other medication. There is currently no information to establish the proportion of patients who use one or the other group of medications to build a base case more in line with the Colombian reality. Additionally, it is not possible to know whether all patients who use an ARA II do so because of intolerance to ACE inhibitors or if the medication was prescribed from the beginning of their pharmacological treatment. Thus, we deemed that the best way to present this cost was to consider both groups of medications.

Cardiovascular disease is one of the top five causes of death in the world and it represents an important burden for the health systems. Our study provides comparative evidence about costs and effectiveness very useful for Colombian and Latin American health authorities at micro and macro levels.
